# Intraoperative Visualization of Bilateral Thrombosis in the Posterior Inferior Cerebellar Artery Apparent in the Telovelomedullary Segment

**DOI:** 10.1155/2014/247652

**Published:** 2014-09-28

**Authors:** Edin Nevzati, Bawarjan Schatlo, Ali-Reza Fathi, Javier Fandino, Carl Muroi

**Affiliations:** Department of Neurosurgery, Kantonsspital Aarau, Tellstraße, 5001 Aarau, Switzerland

## Abstract

Unilateral posterior inferior cerebellar artery (PICA) thrombosis is frequent. However, bilateral PICA thrombosis is rare. Herein we report about an intraoperative visualization of a bilateral thrombosis of the telovelomedullary segment of the PICA. A 74-year-old woman was admitted to our department on day two of a bilateral PICA thrombosis with developing cerebellar infarction. Her Glasgow Coma Scale score dropped from 15 to 13, and cranial computed tomography revealed compression of the fourth ventricle with consecutive occlusive hydrocephalus. After the insertion of an external ventricular drainage, the patient underwent urgent suboccipital decompressive craniectomy with removal of infarcted cerebellar tonsils, which allowed the bilateral visualization of the thrombosed telovelomedullary segments. The surgical access may offer surgical therapeutic options in a hyperacute occlusion, such as thromb-/embolectomy or bypass procedures.

## 1. Introduction

Unilateral posterior inferior cerebellar artery (PICA) thrombosis is the most frequent type of cerebellar infarction and accounts for approximately 2% of all ischemic strokes [1]. Depending on the site of occlusion, clinical manifestation varies widely, from lack of symptoms to a severe medullary and cerebellar picture. Complete Wallenberg's syndrome is found in up to 15% of ischemic strokes in the PICA distribution [2]. Due to its small diameter (about 2.5 millimeters) [3], tomographic imaging of the PICA may be flawed by artifacts and limited by uncertainties. Therefore, in addition to the infarct distribution, angiography is the modality of choice for the diagnosis of occlusion. A macroscopic surgical visualization of bilateral acute PICA thrombosis has not been reported to date. Herein we report about an intraoperative visualization of a bilateral thrombosis of the telovelomedullary segment of the PICA. Surgical and clinical management strategies are briefly discussed.

## 2. Case Report

**Figure 1:**
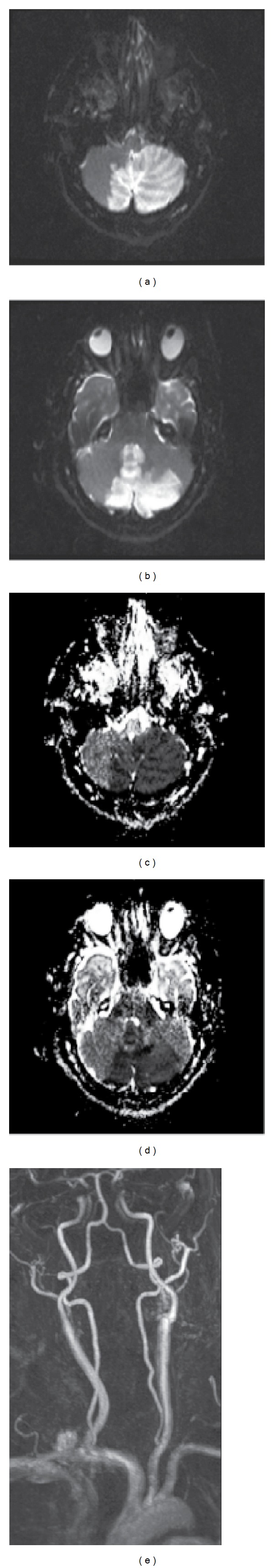
Cranial MRI performed on day two after bilateral PICA infarct. Diffusion-weighted images (a)-(b) and diffusion coefficient maps (c)-(d) revealing fresh infarcts. The 3-dimensional time-of-flight magnetic resonance angiogram shows patency of both vertebral arteries, whereas absence of flow-related signal in both inferior posterior cerebellar arteries is demonstrated (e).

A 74-year-old woman was initially seen in the emergency department of a peripheral hospital presenting with acute onset of headache, dizziness, and limb weakness. Her medical history revealed cerebrovascular risk factors, that is, long year tobacco abuse, hypertension, and dyslipidemia. The patient was clearly conscious with a Glasgow Coma Scale (GCS) of 15 but had a left-sided Wallenberg's syndrome, right brachial weakness (M4), left central facial paresis, left lateral gaze nystagmus, dysarthria, bilateral dysmetria on finger-to-nose and heel-to-shin tests, and truncal ataxia so severe that the patient could not stand. National Institute of Health Stroke Scale (NIHSS) score was 9. Initial cranial computed tomography (CT) did not show any signs of cerebellar pathologies. The patient was treated with aspirin and statin as an embolic infarction could not be ruled out. Electrocardiography showed intermittent atrial fibrillation with spontaneous conversion to sinus rhythm on the second day of hospitalization. Transthoracic echocardiography could not identify any atrial thrombi or valvular pathologies. On day two after onset, a magnetic resonance imaging (MRI) scan was performed. Diffusion-weighted imaging showed left lateromedullar hyperintensity as well as an infarction of both cerebellar tonsils (Figures 1(a)–1(d)). Moreover, the distribution of the bilateral PICA infarct corresponded to type B according to Kang et al. [4], with a complete infarct in the left inferior cerebellar hemisphere and a less extensive infarct in the right hemisphere. Both PICAs could not be visualized in the MR angiography, most likely due to the bilateral occlusion of the vessels. The vertebral artery showed an inconspicuous topographic course on both sides, without any signs of occlusion (Figure 1(e)).

**Figure 2:**
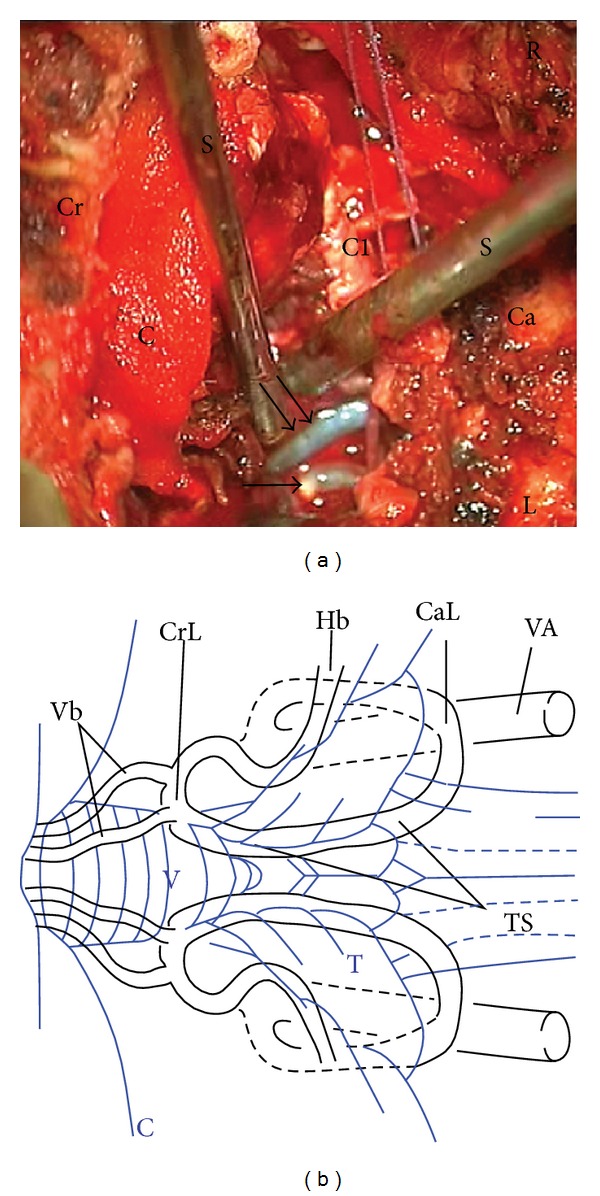
(a) Intraoperative photograph, showing readily visible bilateral PICAs, after the removal of the tonsils. The arteries were pulseless, rigid, and dark. A seemingly calcified thrombus was visible in left PICA (arrow). The right thrombosed PICA was bluish (double arrow). C indicates cottonoid placed over the remaining occipital bone; C1: remaining parts of the C1 arch; Cr: cranial; Ca: caudal; L: left; R: right; S: suction. (b) Schematic drawing of the PICA. C indicates cerebellum; CaL: caudal loop of the PICA; CrL: cranial loop of the PICA; Hb: hemispheric branch; T: cerebellar tonsils, Ts: telovelomedullary segment; V: vermis; VA: vertebral artery; Vb: vermian branches.

On day two after stroke, the patient was transferred to the neurosurgical department due to incessant vomiting, progressive dysphagia, and a decreased level of consciousness. Accordingly the GCS dropped from 15 to 13. Cranial CT revealed an ischemic cerebellar edema with compression of the fourth ventricle and consecutive occlusive hydrocephalus. After the insertion of an external ventricular drainage (EVD) in the anterior horn of the right ventricle, the patient underwent urgent suboccipital decompressive craniectomy. The surgery was performed in prone position. A paramedian right-sided suboccipital craniectomy—crossing the midline to the contralateral side—was performed, combined with a partial C1 laminectomy. After the opening of the dura, clearly infarcted cerebellar tissue was removed until the space occupying effect was relieved. This procedure allowed a good visualization of the thrombosed bilateral PICA segments (Figure 2).

After about two weeks of disabling vertigo the patient was mobilized and had an otherwise uneventful recovery. At discharge the patient had a GCS of 15 and the reaction level scale score was 2. On late follow-up two months following stroke, the patient had a Modified Ranking Scale score of 2.

## 3. Discussion

As the PICA usually originates directly from a single vertebral artery, bilateral infarcts in the PICA distribution are rare. The clinical presentation is similar to that of unilateral PICA infarction [4]. Early diagnosis of cerebellar stroke with appropriate neurological examination, awareness of cerebellar stroke symptoms, and knowledge of brain imaging is crucial, as mortality is as high as 40% in misdiagnosed cerebellar infarcts [5]. The pathogenesis of bilateral PICA distribution infarction has not been sufficiently defined to date [4]. The most frequently postulated mechanism is that a dominant PICA gives rise to both medial branches and their occlusion is due to an embolus or atherosclerotic occlusion in the context of cerebrovascular disease [4, 6–8].

Given the rarity of bilateral PICA thrombosis with subsequent downstream cerebellar infarction, optimal medical and surgical management remains controversial. For embolic occlusion, thrombolysis in the hyperacute phase may be reasonable. At the time of admission our patient had a stroke onset more than 48 hours previously, so thrombolysis was not considered. Few long-term follow-up data are available regarding bilateral cerebellar infarction of PICA distribution. Overall outcome seems to be favorable in this stroke subgroup without primary brainstem involvement. Nonetheless, a bilateral cerebellar infarction may lead to progressive swelling, with brainstem compression and occlusive hydrocephalus. In this case, surgical decompression is warranted.

Pfefferkorn et al. observed no difference in outcomes between patients treated with suboccipital decompressive craniectomy for unilateral cerebellar infarction and those treated for bilateral cerebellar infarction, assuming that an infarction with a bilateral distribution should not be considered a contraindication for surgical decompression [9]. Further, early and aggressive treatment of bilateral cerebellar infarction with a suboccipital decompressive craniectomy resulted in favorable clinical outcome, especially when there was no established brainstem infarction [10]. Chen et al. included, in their series of 11 patients treated with decompressive suboccipital craniectomy and temporary ventriculostomy in malignant cerebellar edema after a large infarction, a total of three bilateral cerebellar infarctions. Since neurological improvement could be seen in most patients soon after the first postoperative day, they concluded that this operative procedure may be lifesaving in cases of progressive neurological deterioration while being on medical therapy for large infarctions [11].

Since its first description in 1994 by Tada et al. [8], there has been an increasing number reporting about acute bilateral cerebellar infarctions in the territory of both PICAs. However, this kind of stroke remains still rare. To the authors' knowledge, fewer than forty cases have been reported in the literature so far [4, 6–8, 11–15], and only a fraction of it has been managed operatively. So far we are the first to visualize bilateral thrombosed PICAs in the televelomedullary segment. The ischemic stroke in our case was most likely attributable to a double, simultaneous embolic vessel occlusion by known cerebrovascular risk factors and visible thrombi. Because of the deteriorating clinical presentation, with progressive cerebellar swelling and a consecutive occlusive hydrocephalus, an immediate treatment of hydrocephalus with an EVD and surgical brainstem decompression by a suboccipital decompressive craniectomy with removal of necrotic cerebellar tonsils was performed. The PICA was exposed along the entirety of the according segment. Thus surgical access, which is readily obtained after sacrifice of the cerebellar tonsils, may offer surgical therapeutic options in hyperacute PICA occlusion. This potentially allows for microsurgical emergency procedures in case of failure of endovascular revascularisation or concomitance of contraindications in hyperacute PICA occlusion. These surgical revascularizations may include open thromb-/embolectomy [16], occipital artery-PICA end-to-side anastomosis [17, 18], and/or PICA/PICA side-to-side anastomosis [19] depending on the location and extension of thromboembolic occlusion. In case of vital cerebellar tonsils the PICA's televelomedullary segment may be approached by dissection of the cerebellomedullary fissure. Although emergency bypass procedures or embolectomy has been described for anterior circulation stroke [16, 20], the applicability for cerebellar stroke is certainly questionable and remains to be elucidated.
